# Investigation of Human Albumin-Induced Circular Dichroism in Dansylglycine

**DOI:** 10.1371/journal.pone.0076849

**Published:** 2013-10-16

**Authors:** Fernanda S. Graciani, Valdecir F. Ximenes

**Affiliations:** 1 Departamento de Química, Faculdade de Ciências, Universidade Estadual Paulista, Bauru, SP, Brazil; 2 Departamento de Análises Clínicas, Faculdade de Ciências Farmacêuticas, Universidade Estadual Paulista, Araraquara, SP, Brazil; Aligarh Muslim University, India

## Abstract

Induced circular dichroism (ICD), or induced chirality, is a phenomenon caused by the fixation of an achiral substance inside a chiral microenvironment, such as the hydrophobic cavities in proteins. Dansylglycine belongs to a class of dansylated amino acids, which are largely used as fluorescent probes for the characterization of the binding sites in albumin. Here, we investigated the ICD in dansylglycine provoked by its binding to human serum albumin (HSA). We found that the complexation of HSA with dansylglycine resulted in the appearance of an ICD band centred at 346 nm. Using this ICD signal and site-specific ligands of HSA, we confirmed that dansylglycine is a site II ligand. The intensity of the ICD signal was dependent on the temperature and revealed that the complexation between the protein and the ligand was reversible. The induced chirality of dansylglycine was susceptive to the alteration caused by the oxidation of the protein. A comparison was made between hypochlorous acid (HOCl) and hypobromous acid (HOBr), and revealed that site II in the protein is more susceptible to alteration provoked by the latter oxidant. These findings suggest the relevance of the aromatic amino acids in the site II, since HOBr is a more efficient oxidant of these residues in proteins than HOCl. The three-dimensional structure of HSA is pH-dependent, and different conformations have been characterised. We found that HSA in its basic form at pH 9.0, which causes the protein to be less rigid, lost the capacity to bind dansylglycine. At pH 3.5, HSA retained almost all of its capacity for binding to dansylglycine. Since the structure of HSA at pH 3.5 is expanded, separating the domain IIIA from the rest of the molecule, we concluded that this separation did not alter its binding capacity to dansylglycine.

## Introduction

Human serum albumin (HSA), the predominant protein in body fluids, has many physiological functions due to its high capacity as a carrier and as a reservoir for a large diversity of endogenous and exogenous molecules [1]. HSA has three globular domains, each of which is divided into two subdomains. They are called subdomains IA, IIA, IB, IIB, IIIA and IIIB. The major binding sites for pharmaceuticals, referred to as site I and site II, are located in the hydrophobic cavities of the subdomains IIA and IIIA, respectively [2]–[4]. Site I is usually referred to as the warfarin binding site, but also has high affinity for piroxicam, phenylbutazone, etc. Site II, also designated the benzodiazepine binding site, has high affinity for diazepam, ibuprofen, etc. [5]–[8].

The ability of a drug to bind to albumin is of fundamental importance, since it affects its distribution in the body, rate of metabolism, and excretion [9]. The characterization of how a drug binds to albumin is among the several pharmacokinetic determinations performed when a new or modified drug is discovered or synthesised. During the characterisation, parameters such as the binding constant, number of binding sites, and the determination of which binding site has affinity for the studied drug are normally measured [10]. A common approach for characterising the binding sites in albumin is the use of fluorescent dansylated amino acids. These fluorescent probes were proposed by Sudlow and collaborators in 1975, who divided these compounds in two groups: (i) those that bind to the warfarin binding site (site I), which includes dansylcysteic acid, dansyl-L-arginine, dansyl-L-glutamine, and dansylamide; and (ii) those that bind to the benzodiazepine binding site (site II), which includes dansylsarcosine, dansyl-alpha-aminobutiric acid, dansyl-hydroxy-L-proline, and dansyl-L-proline [3], [4]. Another dansylated amino acid that is widely employed for the characterization of binding sites is dansylglycine, but there remain questions about its site specificity [11]–[14].

The wide applicability of dansylated amino acids comes from the fact that their fluorescence quantum yields are significantly increased when bonded to albumin. Moreover, a blue shift in the maximum of the emission wavelength is typically observed. This is a typical phenomenon associated with the change from hydrophilic to hydrophobic medium (e.g., the displacement of the probes from the aqueous medium to the hydrophobic cavity inside the protein) [15]. Therefore, the decrease in the fluorescence of the protein-dansyl amino acid complex caused by the addition of a specific drug is the analytical parameter for detection of its binding site [16].

Another phenomenon resulting from the binding of many compounds to HSA is the generation of a new circular dichroism signal for the protein-ligand complex. This is known as induced circular dichroism (ICD), or induced chirality, and results from the attachment of the optically inactive substance inside the asymmetric microenvironment, which forms the protein binding site. In other words, ICD occurs when the achiral guest is encircled by a chiral host. This induced chirality has been demonstrated for many pharmaceuticals, including phenylbutazone, diazepam, ketoprofen, warfarin, etc. [17]–[18]. The ICD absorption band is observed in the same, or similar, absorption wavelength for the chromophoric part of the ligand. This is direct evidence of the complexation between the protein and the ligand. Similar to the displacement of the fluorescent probes from the binding sites, competition experiments based on ICD have also been used to characterise binding sites using ligands that are susceptible to ICD [19], [20].

In this report, our goal was to determine the biophysical properties of the interaction of dansylglycine with HSA. The focus of this study is the induction of chirality in dansylglycine and how the ICD signal is affected by changes in the protein resulting from changes in pH, temperature, and oxidation. The results show that dansylglycine is a site II ligand and contribute to the characterization of the site II of HSA.

## Materials and Methods

### Chemicals

Human serum albumin free of fatty acids, warfarin, phenylbutazone, ibuprofen, naproxen, and dansylglycine were purchased from Sigma-Aldrich Chemical Co. (St. Louis, MO, USA). Stock solutions of the pharmaceuticals (10 mM) were prepared in ethyl alcohol. Stock solution of dansylglycine (5 mM) was prepared in 10 mM hydrochloric acid. The protein was dissolved in 50 mM phosphate buffer at pH 7.0 to give a 1 mM stock solution, which was stored at 4°C. Protein concentration was determined by measuring its absorbance at 280 nm (ε_280 nm_ = 37,219 M^–1^cm^–1^) [21] on a Perkin Elmer Lambda 35 UV−visible spectrophotometer (Shelton, CT, USA).

### Fluorescence Experiments: Determination of Quenching and Binding Constants

The fluorescence spectra of HSA were obtained using a Perkin Elmer LS 55 spectrofluorimeter (Shelton, CT, USA) with the following settings: excitation at 295 nm and emission scanning between 310 and 450 nm. The slit widths were 2.5 nm for excitation and 10 nm for emission wavelengths. A 3 mL quartz cuvette with a 10 mm path length and a magnetic stirrer were used during the measurements. The fluorescence spectra were automatically corrected for emission. Fluorescence quenching experiments were performed by the addition of varying amounts of dansylglycine (0–35 µM) to the protein (5 µM) in 50 mM phosphate buffer, pH 7.0, at different temperatures. The mixtures were incubated for 5 min before the measurements were taken. The fluorescence intensities, measured at 343 nm, were corrected for the inner filter effect caused by attenuation of the excitation and emission signals resulting from the absorption of the quencher using the following equation (Eq. 1).
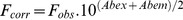
(1)Where *F_corr_* and *F_obs_* are the corrected and observed fluorescence intensities, respectively; *Ab_ex_* and *Ab_em_* are the absorptions of the mixture at excitation and emission wavelengths, respectively [22]. Absorbances were measured using a Perkin Elmer Lambda 35 UV−visible spectrophotometer (Shelton, CT, USA).

### Fluorescence Displacement Experiments

For displacement assays using the fluorescent probe dansylglycine, the spectrofluorimeter was adjusted to the following parameters: excitation at 340 nm and emission in the range 400–650 nm; slit widths 2.5 nm for excitation and 10 nm for emission wavelengths in a 3 mL quartz cuvette with a 10 mm path length and a magnetic stirrer. The experiments were performed by the addition of varying amounts of the pharmaceuticals (0–20 µM, final concentrations) to a mixture of 5 µM HSA and 5 µM dansylglycine in 50 mM phosphate buffer at pH 7.0. After addition of the pharmaceuticals, the mixtures were incubated for 5 min at 25°C before the measurements.

### Circular Dichroism Experiments

Circular Dichroism (CD) spectra were recorded with a Jasco J-815 spectropolarimeter (Jasco, Japan) equipped with a thermostatically controlled cell holder. The spectra were obtained in duplicate with 1 nm step resolution, response time of 1 s and scanning speed of 20 nm/min. The near-UV CD spectra were recorded at a protein concentration of 15 or 30 µM and increasing concentrations of dansylglycine and pharmaceuticals over a wavelength range of 250–450 nm at 25°C. A 3 mL quartz cuvette with a 10 mm path length and a magnetic stirrer were used for the measurements. The baseline (50 mM phosphate buffer) was subtracted from all measurements. For the displacement studies, the spectra were recorded 5 min after addition of the pharmaceuticals to the complex of HSA-dansylglycine.

For assessment of the thermal denaturation of HSA, the ellipticity was recorded at a protein concentration of 2 µM using a 2 mm path length quartz cuvette. The thermal denaturation curve was determined by monitoring the changes in the CD signal at 222 nm in the temperature range 20–95°C with a heating rate of 1°C/min. The temperature was measured at 5°C intervals, holding for 2 min before the measurements at each temperature, and then reversed to 20°C following the same protocol. A Jasco PTC-348 thermostat (Tokyo, Japan) was used for the thermal denaturation studies.

### Oxidation of HSA by Hypohalous Acid

Hypochlorous acid (HOCl) was prepared by diluting a 5% concentrated solution, and the concentration was determined spectrophotometrically after dilution in 0.01 M NaOH at pH 12 (ε292 nm = 350 M^–1^cm^–1^) [23]. HOCl was diluted to give a working solution of 100 mM in water. HOBr was synthesised by combining 100 mM HOCl and 200 mM NaBr in water [24]. The reaction mixtures contained 30 µM HSA and 600 or 1200 µM oxidant in 50 mM sodium phosphate buffer at pH 7.0 and 25°C. The reaction mixtures were incubated for 2 h at 25°C and excess oxidants were removed by adding 2 mM methionine and incubated for an additional 5 min. Dansylglycine (30 µM) was added and the mixture incubated for 5 min before measuring the induced ellipticity.

## Results and Discussion

### Determination of the Binding Constant for Dansylglycine

**Figure 1:**
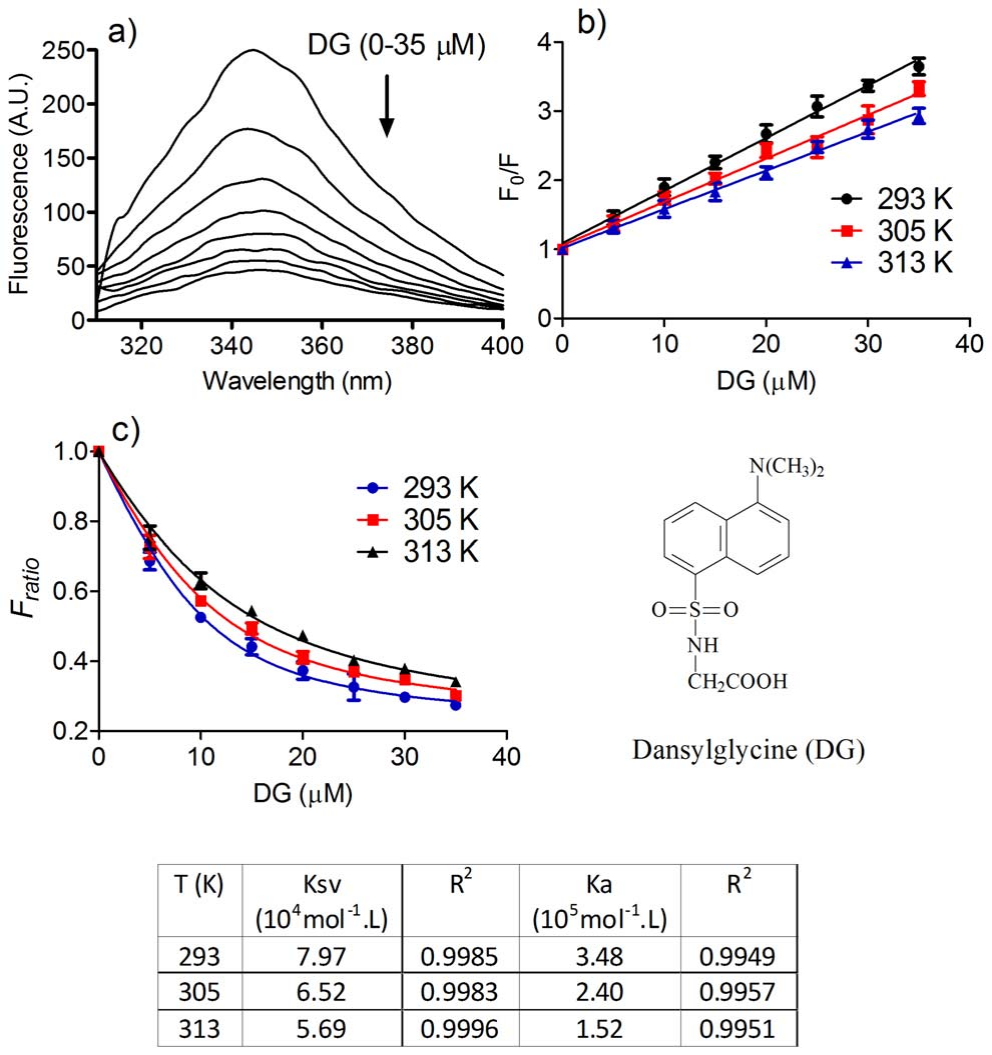
Quenching of the intrinsic fluorescence of HSA by dansylglycine and determination of binding constant. (**a**) Emission spectra of HSA (5 µM; λ_ex_ = 295 nm) in the presence of dansylglycine (DG; 0–35 µM). (**b**) Stern-Volmer plots at different temperatures. (**c**) Non-linear fitting for determination of the binding constant (Ka). **(Table)** Stern-Volmer and binding constants at different temperatures. The results are the average and SD of experiments performed in triplicate.

Initially, we studied the biophysical characteristics of the association between dansylglycine and HSA. The analytical approach we used for measuring the interaction was fluorescence quenching, a phenomenon associated with a decrease in the steady state fluorescence and/or its lifetime caused by collisional deactivation (dynamic quenching) and formation of a ground-state complex (static quenching), among other mechanisms [15]. The results in Figure 1a show that the fluorescence intensity of HSA was strongly decreased by the addition of dansylglycine. In these experiments, aliquots of dansylglycine were added to 5 µM HSA and the mixture equilibrated for 5 min before the measurements were taken. The intrinsic fluorescence of the protein was almost completely quenched by adding 7 molar excess dansylglycine, and a linear relationship was obtained using the Stern-Volmer equation (Eq. 2, Figure 1b). It is worth noting that in order to eliminate the inner filter effect caused by the absorbance of the ligand, the fluorescence intensities were corrected before the application of the Stern-Volmer analysis.
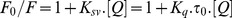
(2)Where *F_0_* and *F* are the fluorescence intensity in the absence and presence of the quencher, respectively; *K_sv_* is the Stern-Volmer constant; *K_q_* is the bimolecular quenching constant; *τ_0_* is the average lifetime of fluorophore in the absence of quencher and *[Q]* is the concentration of the quencher [15]. *K_q_*, which was calculated from *K_sv_* (7.97×10^4^ M^−1^, Figure 1) and assuming *τ_0_*∼5×10^−9^ s for the tryptophan in the protein [15], resulted in ∼1.6×10^13^ M^−1^s^−1^. As this value is higher than the maximum collision quenching constant ∼10^10^ M^−1^s^−1^ [25], a static process can be attributed as responsible by the fluorescence quenching of HSA.

Confirmation that the fluorescence quenching resulted from a static process, such as the formation of a ground state complex between dansylglycine and HSA, was obtained by observing the effect of temperature on the Stern-Volmer constant. It is well-established that the interaction between the ligand and the protein is usually weakened by increasing temperature [15]. From the results presented in Figure 1, it can be concluded that this was the case for the interaction between HSA and dansylglycine, reinforcing the proposal for a complexation between these molecules.

Taking in account the above results, the binding affinity (association constant, *K_a_*) between HSA and dansylglycine was obtained using a nonlinear fitting (Eq. 3).
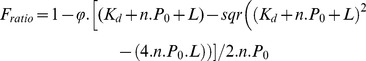
(3)


In this equation, *F_0_* is the fluorescence in the absence of ligand; *F* is the fluorescence in the presence of ligand; *F_ratio_* (*F/F_0_*) is the observed fluorescence ratio; φ is the fluorescence ratio change amplitude (1-*F_ratio_*_∞_); *F_ratio∞_* is the ratio at an infinite concentration of the ligand; *P_0_* is the protein concentration; *L* is the concentration of added ligand; *K_d_* is the dissociation constant; and *n* is the number of binding sites [26]. Here, since a 1∶1 complex was assumed, *n* was set as 1. This value was assumed based in a previously reported determination [14]. Therefore, only *K_d_* and *Φ* were treated as the fitting parameters in the nonlinear least-squares analysis (GraphPad Prism version 5.00 for Windows, GraphPad Software, San Diego California USA). *K_a_* was calculated as *1/K_d_*.

It is worth noting that this mathematical model has an independent variable: the concentration of the added ligand *L*. It is more complex than the usual and widely applied non-quadratic model that assumes that *L = L_F_* (ligand free in equilibrium), but more appropriate when the concentration of the ligand is close to the concentration of the protein [27], which was the case in our studies. The value obtained for *K_a_* (Figure 1c and Table in Figure 1) is in agreement with a previously reported estimation [14].

### Displacement of Dansylglycine by Specific Ligands

**Figure 2:**
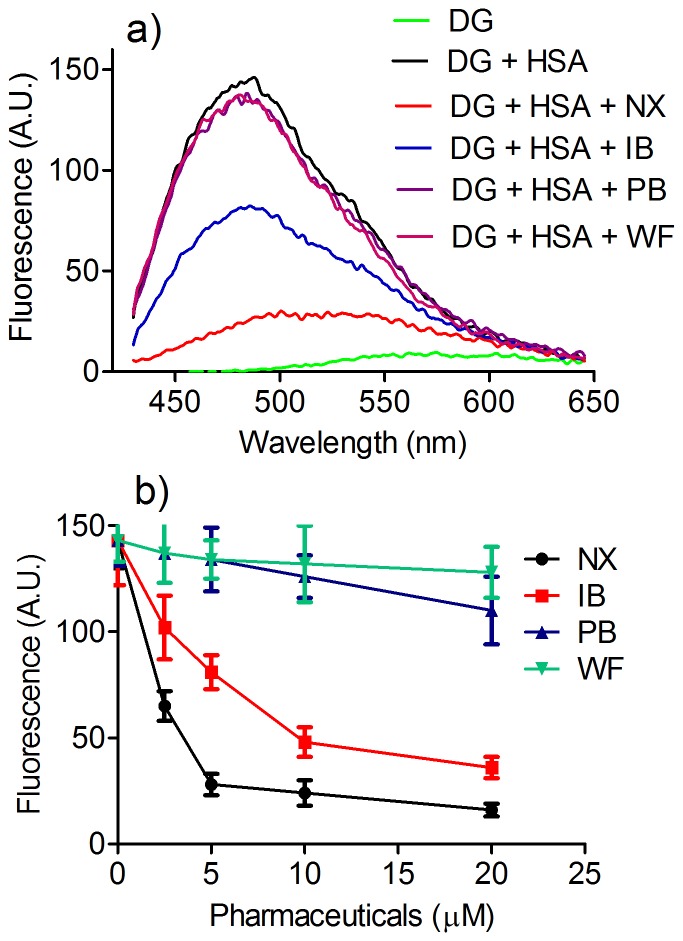
The effect of HSA on dansylglycine (DG) fluorescence and displacement by site I and II specific ligands. (**a**) The mixtures consisted of 5.0 µM HSA, 5.0 µM DG, and 5 µM of naproxen (NX), ibuprofen (IB), phenylbutazone (PB) or warfarin (WF). (**b**) Concentration dependent displacement. The results are the average and SD of experiments performed in triplicate.

The protein-ligand association was also demonstrated by the increased fluorescence efficiency and shift to a lower wavelength when HSA was added to dansylglycine (Figure 2a). This indicates that dansylglycine was subjected to a more hydrophobic microenvironment, i.e. the binding site of HSA. In the sequence, the addition of known specific site I and II ligands was used to determine the binding site of dansylglycine. The results, depicted in Figure 2b, show that warfarin and phenylbutazone (site I ligands) [5]–[8] were ineffective in the displacement of dansylglycine from HSA. On the other hand, the site II ligands ibuprofen and naproxen were efficient in its displacement. It is worth noting that this conclusion was based on the decrease of the dansylglycine fluorescence caused by its removal from the binding site of the protein. These results confirm that dansylglycine is a site II ligand, as has been demonstrated [12]–[14], and not site I ligand, as has also been suggested [11].

### HSA-induced Ellipticity in Dansylglycine

**Figure 3:**
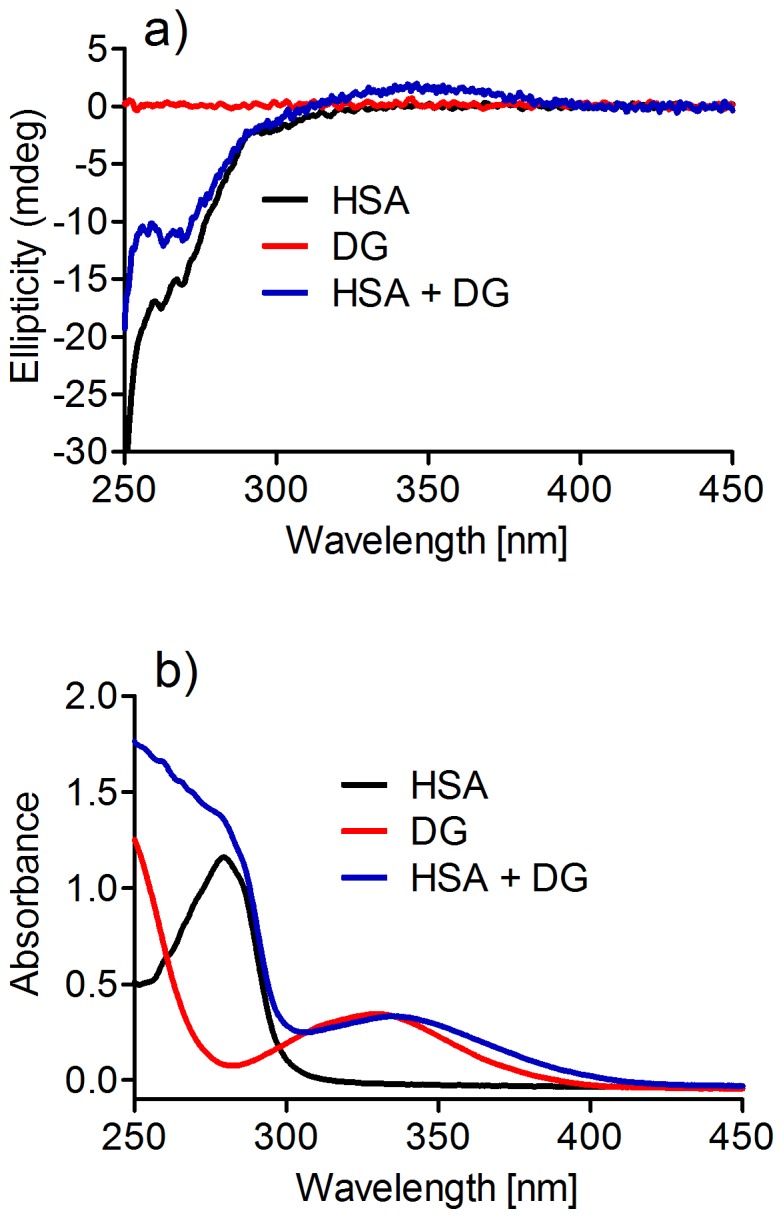
HSA-Induced ellipticity in dansylglycine. (**a**) Near-UV-CD and (**b**) UV-Vis spectra of dansylglycine in the presence or absence of HSA. The mixtures consisted of 30 µM HSA and 30 µM DG.

Figure 3 shows the near-UV CD spectra (a) and the UV absorption (b) of HSA and dansylglycine. Albumin has a characteristic near-UV-CD spectrum below 300 nm, with two minima at 262 and 269 nm, which are attributed to aromatic residues and disulfide bond [28]. Dansylglycine, which absorbs with a maximum at 330 nm, is not an optically active molecule and, obviously, it is not able to produce a circular dichroism signal. However, its complexation with HSA resulted in the appearance of a circular dichroism spectrum centred at 346 nm (Figure 3a). This induced ellipticity in dansylglycine is additional evidence of the association between HSA and the fluorescent probe, and can be explained by the binding of dansylglycine to the asymmetric environment inside the albumin binding site.

**Figure 4:**
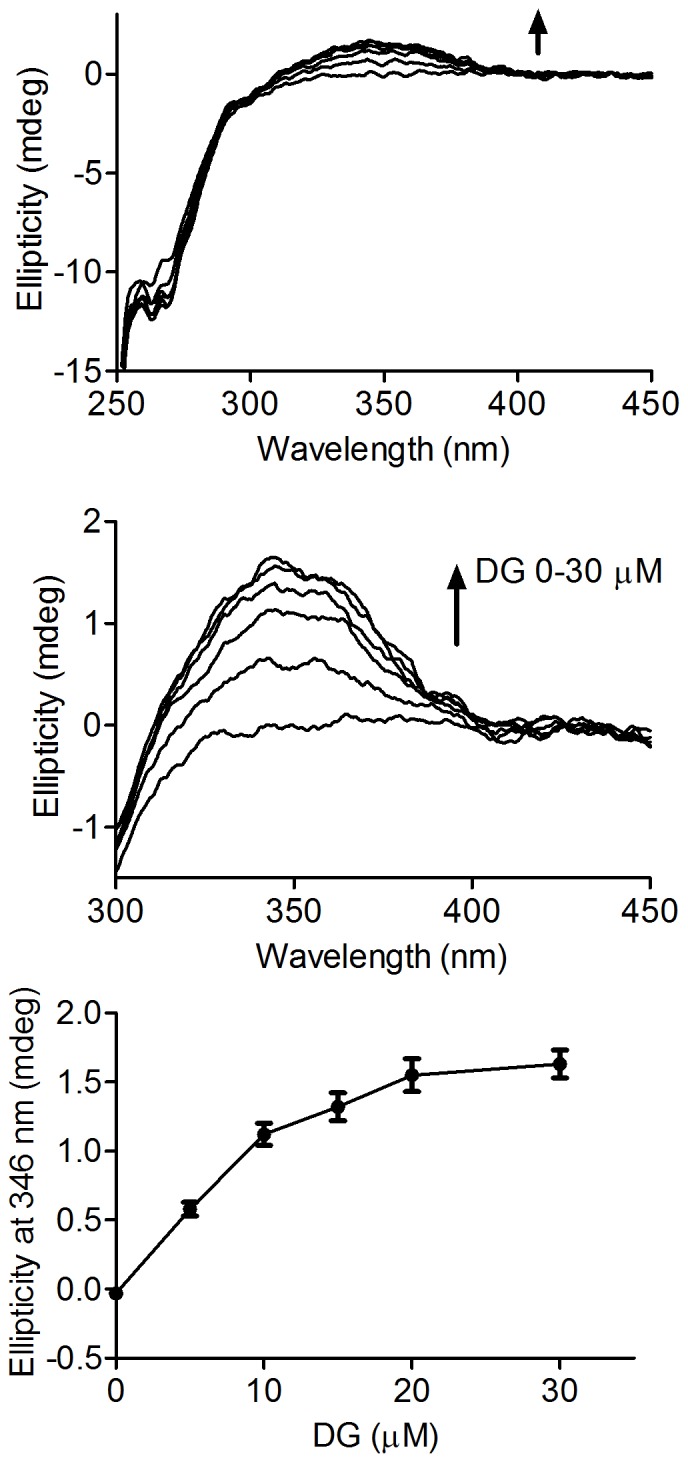
Induced ellipticity as a function of dansylglycine concentration. The mixtures consisted of 15 µM HSA and increasing concentration of DG, as indicated. The results are the average and SD of experiments performed in triplicate.

Binding of dansylglycine to HSA was also responsible for the slight red-shift in the absorption spectrum of dansylglycine (Figure 3b). In fact, it is well-known that the energy difference between ground and excited states is dependent on the environment. Therefore, inside the hydrophobic cavity of the protein, the ground state of dansylglycine was destabilised when compared to the aqueous medium, which explains the lower energy difference between the ground and excited states. Corroborant with this, a red-shift in the absorption wavelength is typical of many molecules when complexed with proteins [29], [30]. Figure 4 shows the addition of dansylglycine to HSA and the concentration-dependent increase in the ICD band. The maximum in the ICD signal at 346 nm was reached at almost equimolar concentrations of HSA and dansylglycine, which is an indication of one binding site for this fluorescent probe, as previously reported [14].

**Figure 5:**
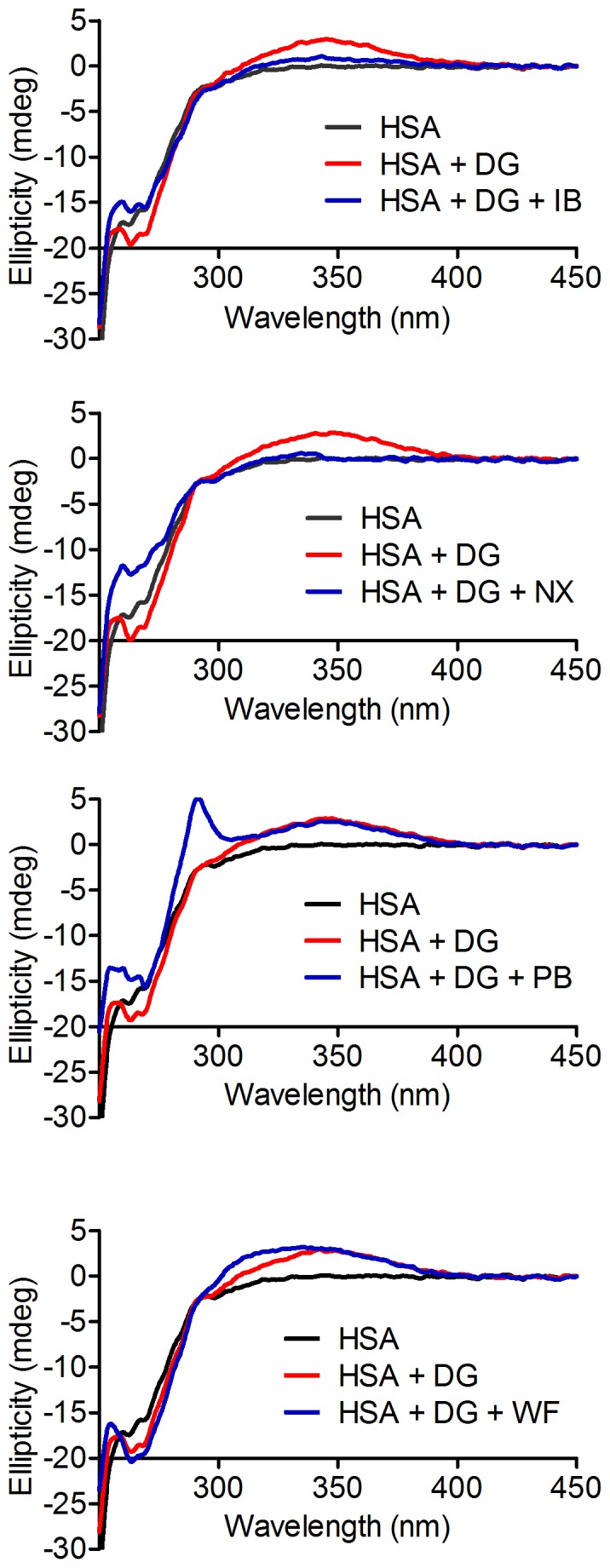
Displacement of dansylglycine (DG) from HSA by site I and II ligands: circular dichroism studies. The mixtures consisted of 30 µM HSA, 30 µM DG, and 30 µM of naproxen (NX), ibuprofen (IB), phenylbutazone (PB), or warfarin (WF).

The ICD of dansylglycine was used to confirm its binding at the site II of HSA. The results, depicted in Figure 5, show that ibuprofen and naproxen (site II), but not phenylbutazone and warfarin (site I), were effective in the displacement of dansylglycine from HSA, causing a decrease in its chirality. These findings open up the possibility of using the measurements of the ICD signal for dansylglycine for evaluating the binding sites for new ligands that bind albumin. This technique could be useful when the fluorescence-displacement assay (Figure 2) is not applicable, such as when the studied ligand fluoresces at the same wavelength as the probe. It is worth noting that ibuprofen and warfarin were also susceptible to induced chirality, but in different wavelengths than dansylglycine (Figure 5).

### Effect of Temperature on Induced Ellipticity in Dansylglycine

**Figure 6:**
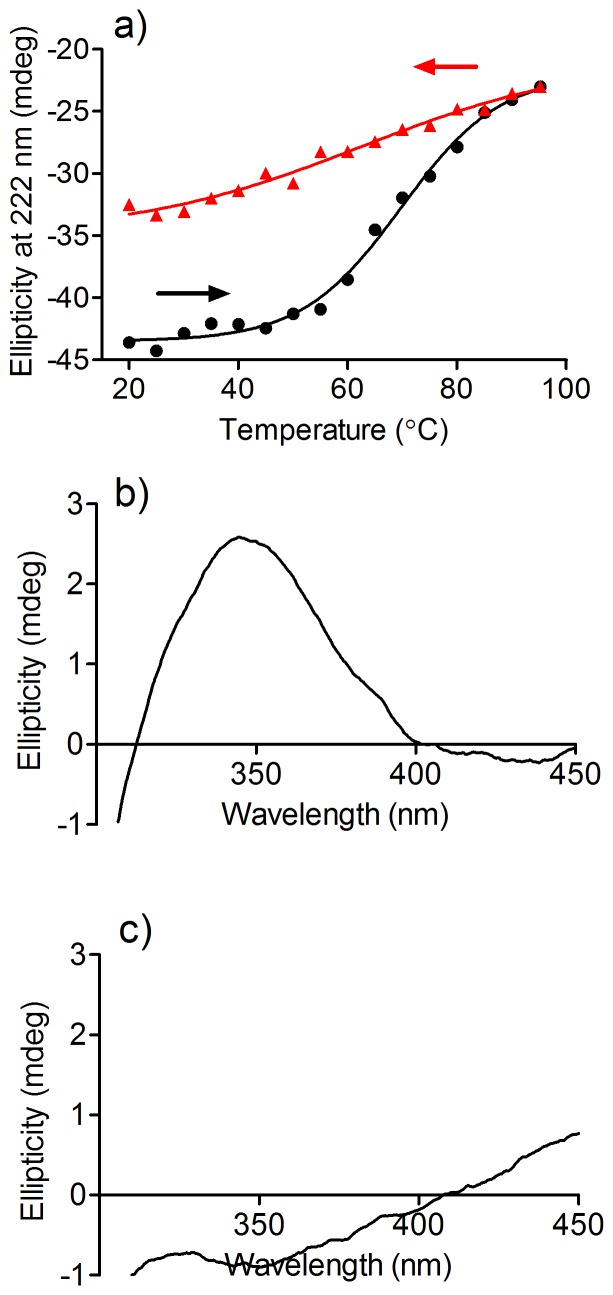
Temperature-induced denaturation of HSA and its effect on ICD of dansylglycine. (**a**) Denaturation curve of HSA. The temperature was increased at 1°C/min, equilibrated at the target temperature for 2 min before each measurement and reversed following the same protocol; (**b**) DG was added before denaturation and the ICD spectrum measured at 25°C; (**c**) DG was added after denaturation and the ICD spectrum measured at 25°C.

Since proteins are susceptible to thermal denaturation, we also studied the effect of temperature on the ICD for dansylglycine. Initially, the denaturation of HSA was studied by increasing the temperature of the protein solution from 20 to 95°C. By plotting the ellipticity of HSA at 222 nm, a parameter of the alpha-helix content in a protein, a typical heat denaturation curve was obtained (Figure 6a) with the transition midpoint (Tm) at 69. 8°C, which is close to a recently reported predicted temperature [31]. Figure 6a also shows that denaturation was an irreversible process, since the alpha-helix content did not return to the same level when the temperature was decreased to 20°C.

**Figure 7:**
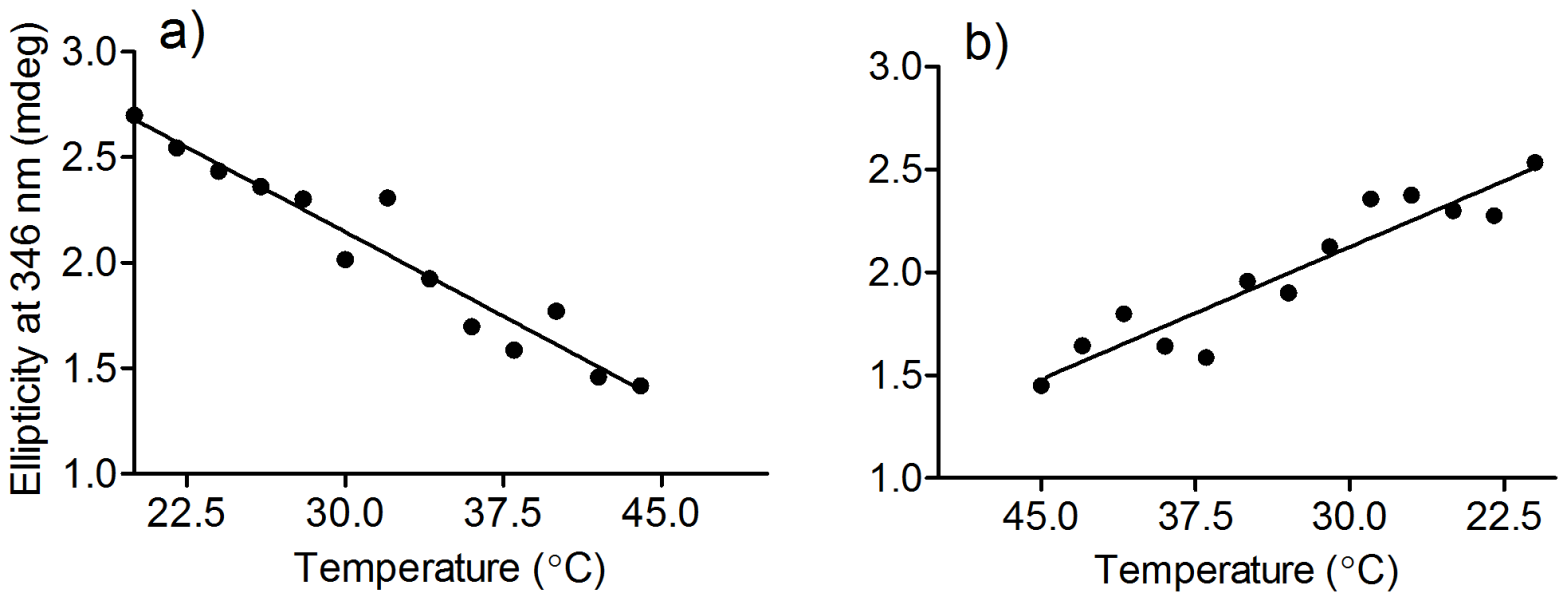
Temperature-dependent reversibility of ICD in dansylglycine. The mixtures consisted of 30 µM HSA and 30 µM DG. (**a**) The temperature was increased from 20°C to 45°C and (**b**) returned to 20°C at a rate of 0.5°C/min.

To evaluate the effect of the denaturation on the binding of dansylglycine, the next step was the addition of the fluorescent probe before and after denaturation of the protein. From the results, displayed in Figure 6b and 6c, it can be concluded that the binding capacity of HSA was completely lost in the denatured form, indicating the binding sites were degraded. However, Figure 6 also shows that the alteration of the alpha-helix content was minimal from 20°C to 45°C, demonstrating that the secondary and tertiary structures of the protein were not changed in this range. Therefore, we used this temperature range for evaluation of the alteration of ICD of dansylglycine, since it could represent changes in the binding affinity as a consequence of weakened interaction between the protein and the fluorescent ligand, rather than protein denaturation. In these experiments, the circular dichroism spectrometer was adjusted to increase the temperature from 20°C to 45°C and then return to 20°C at a rate of 0.5°C/min. The results are depicted in Figure 7 and show that the ICD signal decreased linearly over this temperature range and returned to the original value when the temperature was returned to 20°C. This result provides additional evidence of the reversibility of the binding of dansylglycine to HSA. Moreover, it represents an experimental ICD evidence of the well-accepted temperature-dependence of the binding constant [26].

### Effect of HSA Oxidation on ICD in Dansylglycine

**Figure 8:**
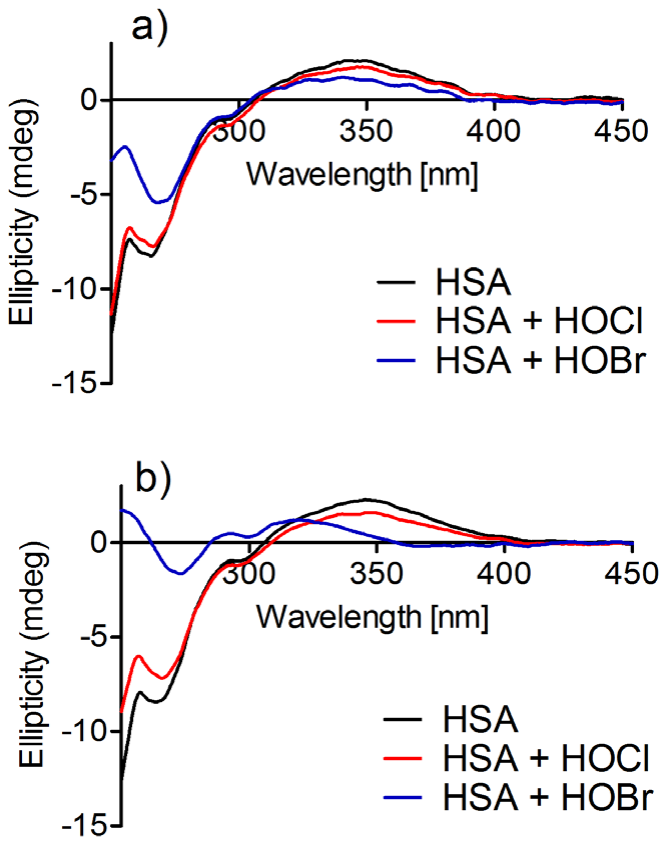
Effect of oxidation on HSA-induced chirality. The mixtures consisted of 30 µM HSA and (**a**) 600 µM or (**b**) 1200 µM of the oxidants. Dansylglycine (30 µM) was added after oxidation.

We found that the induced chirality of dansylglycine could be used to investigate alterations in the structure of HSA resulting from its oxidation. To demonstrate this, the protein was oxidised using hypochlorous acid (HOCl) and hypobromous acid (HOBr). Excess oxidants were depleted by adding methionine, and then dansylglycine was added and the ICD spectra recorded. It is well-established that these chemicals are able to oxidise amino acid residues in proteins resulting in the formation of carbonylated residues, the depletion of intrinsic fluorescence, aggregation, etc., which alter the secondary and tertiary structures of the proteins [32]–[34]. The ICD of dansylglycine was also reduced in oxidised HSA when compared to the native protein (Figure 8). The loss of induced chirality was still more significant when the protein was oxidised by HOBr, an oxidant that is more effective in the depletion of the intrinsic fluorescence of proteins [35], [36], a phenomenon that is related to oxidation of aromatic amino acid residues [37]. These findings suggest that amino acid residues, such as phenylalanine and tyrosine, located in site II of HSA, could play an important role in the development of the chiral microenvironment responsible for the induction of chirality in dansylglycine. In agreement with this, Ryan and collaborators recently co-crystallised HSA with dansylated amino acids specific for site II and elucidated its three-dimensional structure, and found that the dansyl group occupies a position between the side-chains of Asn-391 and Phe-403 on one side and Leu-453 on the other. Moreover, the oxygen atom on one side of the SO_2_ of the dansyl group forms a hydrogen bond with the side-chain of Tyr-411 [38]. Therefore, the stronger capacity of HOBr to act as an oxidant of aromatic residues, and the importance of Phe-403 and Tyr-411 in the interaction of dansylated amino acids with the site II of HSA, could explain the higher efficacy of this oxidant in the impairment of binding and induction of ellipticity in dansylglycine.

### Effect of pH on ICD in Dansylglycine

**Figure 9:**
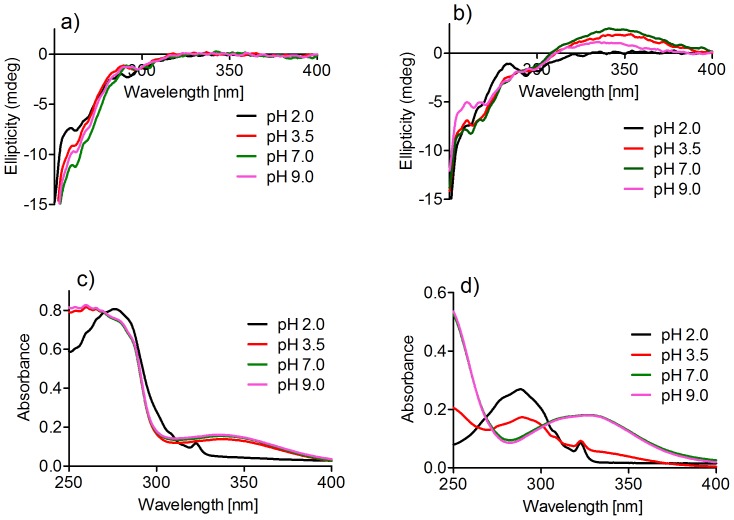
Effect of pH on HSA-induced chirality in dansylglycine. (**a**) HSA (30 µM) was incubated at various pHs. (**b**) CD spectra after addition of 30 µM dansylglycine. (**c**) Absorbance spectra after addition of 30 µM dansylglycine. (**d**) Absorbance spectra of 30 µM dansylglycine alone.

It is well-established that the three-dimensional structure of HSA is pH-dependent, and several conformations have been characterised. Among them, the normal form (N) at pH 7.0, basic form (B) at pH above 8.0, and the fast-migrating form (F) at pH below 4.3. These forms are characterised by changes in the proportions of the alpha-helix and beta-sheet content, and also in their tertiary structure [39]. The decrease in pH induces an expansion of the protein and the displacement of the domain IIIA, which reaches maximum displacement at about pH 2.5. On the other hand, the basic form is characterised by the loss of rigidity, which affects the N-terminal region of the protein [40]. We incubated HSA for 2 h at pHs 2.0, 3.5, 7.0, and 9.0. Then, the near-UV CD spectra were recorded. The results, depicted in Figure 9a, show the alteration in the near-UV CD, which is related to the tertiary structure of HSA, at the various pHs. These results agree with previous results [40]. In summary, both the decrease and increase in pH caused an increase in the ellipticity at the minima 262 and 268 nm, which has been attributed to the loss of asymmetry around disulfide bridges and/or aromatic residues [40], an indication of alterations in the tertiary structure of HSA.

Next, dansylglycine was added and the near-UV CD and absorbance were measured again. From the results shown in Figure 9b, it can be observed that in the basic form HSA partially lost the capacity to induce chirality in dansylglycine. A putative explanation for these findings could be that there was an alteration in the UV-Vis spectrum. For instance, a shift in the maximum of the absorption or a decrease in the molar absorption coefficient of dansylglycine at alkaline pH could explain the decreased ICD signal. However, this was not the case, since no alteration was observed for the absorbance of dansylglycine or for the complex of HSA-dansylglycine when compared the spectra at pHs 7.0 and 9.0 (Figures 9c and 9d). Therefore, we concluded that the basic form of HSA partially lost its capacity for binding to dansylglycine.

At acidic pHs, the induced ellipticity was also progressively lost (Figure 9b). However, under these conditions, an alteration in the UV-Vis properties of dansylglycine could, at least partially, explain the alteration in the ICD. Indeed, at pH 2.0, a blue-shift was observed for the absorbance of dansylglycine alone (Figure 9d) or in the presence of HSA (Figure 9c). It is well-established that, when present, an ICD signal is usually found in the region of absorption of the ligand, hence an alteration in the UV-Vis absorption must reflect this in the ICD spectrum. At pH 2.0, the maximum of absorption of dansylglycine was shifted to 288 nm, which was significantly different when compared to neutral and alkaline pHs, which had maxima at 330 nm. Therefore, the induced CD signal was completely lost at 346 nm, but a new peak at 287 nm (Figure 9b) indicates that dansylglycine was still bonded to HSA.

At pH 3.5, a clear transition in the band of absorption of dansylglycine was observed when compared to the bands for pH 2.0 and 7.0 (Figure 9d). However, at pH 3.5, the absorbance of the HSA-dansylglycine complex was unaltered when compared to the absorbance at pH 7.0 (Figure 9c). In the same direction and in agreement with the alteration in the absorbance of the complex, the ellipticity was also only slightly decreased (Figure 9b). These findings are evidence that at pH 3.5, the protein retained most of its capacity for binding to dansylglycine, which could be considered a new property of the F form of albumin. It is worth noting that, at pH 3.5, HSA was already expanded and separation of the domain IIIA from the rest of the molecule is the main alteration of the protein [40]. Considering that site II is located in the domain IIIA, we can conclude that its separation did not alter its ability to bind dansylglycine.

## Conclusions

The most common experimental approach to measure the association between ligands and proteins is to determine the alterations in the intensity and energy of the absorbance and fluorescence bands of the protein, ligand, or the protein and ligand. These techniques are extremely useful, but some difficulties appear when a superposition of bands occurs. For instance, when the protein and ligand fluoresce or absorb in the same region of the wavelength spectrum. To overcome this limitation, when applicable, the induced chirality can be a very useful parameter since it is directly related to the association phenomenon, and the circular dichroism technique is not affected by the absorbance and/or fluorescence of the protein and ligand. In other words, only if the association takes place does the ICD signal become detectible for the protein-ligand complex. In this report, we have demonstrated that the complexation of HSA with dansylglycine leads to the appearance of a positive CD band centred at 346 nm. Using this CD signal and the effect of specific ligands of site I and II, we confirmed that dansylglycine is an HSA site II ligand, but not a site I ligand, as has been suggested previously. As long as the association between the ligand and protein is responsible by the ICD signal, this analytical parameter can also be useful for the studies of alterations in the protein structure caused by changes in the temperature, acidity, or ionic strength of the medium. In this report, we used this technique to demonstrate that the association between dansylglycine and HSA is completely reversible and dependent on the temperature, with the complexation weakened at higher temperature. We also demonstrated that the alteration in the structure of the protein induced by either the pH of the medium or chemical modification of the amino acids residues had a direct effect on the ICD signal. Therefore, we propose that the ICD of dansylglycine, or of other dansylated amino acids, could be applied to the measurement of the biophysical characteristics of the binding sites of albumin.
